# Growing up in Bradford: protocol for the age 7–11 follow up of the Born in Bradford birth cohort

**DOI:** 10.1186/s12889-019-7222-2

**Published:** 2019-07-12

**Authors:** Philippa K Bird, Rosemary R. C. McEachan, Mark Mon-Williams, Neil Small, Jane West, Peter Whincup, John Wright, Elizabeth Andrews, Sally E Barber, Liam J B Hill, Laura Lennon, Dan Mason, Katy A Shire, Dagmar Waiblinger, Amanda H. Waterman, Deborah A. Lawlor, Kate E. Pickett

**Affiliations:** 1 Born in Bradford, Bradford Institute for Institute for Health Research, Bradford Teaching Hospitals NHS Foundation Trust, Bradford Royal Infirmary, Duckworth Lane, Bradford, BD9 6RJ UK; 2 Leeds Teaching Hospitals NHS Trust, Great George Street, Leeds, LS1 3EX UK; 3 School of Psychology, University of Leeds, Leeds, LS2 9JT UK; 4 Faculty of Health Studies, University of Bradford, Bradford, BD7 1DP UK; 5 Population Health Science, Bristol Medical School, Bristol University, Oakfield House, Oakfield Grove, BS8 2BN UK; 6 Population Health Research Institute, St George’s, University of London, Cranmer Terrace, London, SW17 0RE UK; 7 MRC Integrative Epidemiology Unit at the University of Bristol, Oakfield House, Oakfield Grove, Bristol BS8 2BN UK; 8 Population Health Science, Bristol Medical School, University of Bristol University, Oakfield House, Oakfield Grove, Bristol BS8 2BN UK; 9 Bristol NIHR Biomedical Research Centre, University Hospitals Bristol NHS Foundation Trust, University of Bristol, Oakfield House, Oakfield Grove, Bristol BS8 2BN UK; 10 Department of Health Sciences University of York Seebohm Rowntree Building, University of York, Heslington, York YO10 5DD UK

**Keywords:** Born in Bradford, Birth cohort study, Ethnicity, Mental health, Cardiorespiratory health, Cognitive development, Sensorimotor development, Socio-economic status

## Abstract

**Background:**

Born in Bradford (BiB) is a prospective multi-ethnic pregnancy and birth cohort study that was established to examine determinants of health and development during childhood and, subsequently, adult life in a deprived multi-ethnic population in the north of England. Between 2007 and 2010, the BiB cohort recruited 12,453 women who experienced 13,776 pregnancies and 13,858 births, along with 3353 of their partners. Forty five percent of the cohort are of Pakistani origin. Now that children are at primary school, the first full follow-up of the cohort is taking place. The aims of the follow-up are to investigate the determinants of children’s pre-pubertal health and development, including through understanding parents’ health and wellbeing, and to obtain data on exposures in childhood that might influence future health.

**Methods:**

We are employing a multi-method approach across three data collection arms (community-based family visits, school based physical assessment, and whole classroom cognitive, motor function and wellbeing measures) to follow-up over 9000 BiB children aged 7–11 years and their families between 2017 and 2021. We are collecting detailed parent and child questionnaires, cognitive and sensorimotor assessments, blood pressure, anthropometry and blood samples from parents and children. Dual x-ray absorptiometry body scans, accelerometry and urine samples are collected on subsamples. Informed consent is collected for continued routine data linkage to health, social care and education records. A range of engagement activities are being used to raise the profile of BiB and to disseminate findings.

**Discussion:**

Our multi-method approach to recruitment and assessment provides an efficient method of collecting rich data on all family members. Data collected will enhance BiB as a resource for the international research community to study the interplay between ethnicity, socioeconomic circumstances and biology in relation to cardiometabolic health, mental health, education, cognitive and sensorimotor development and wellbeing.

**Electronic supplementary material:**

The online version of this article (10.1186/s12889-019-7222-2) contains supplementary material, which is available to authorized users.

## Background

Born in Bradford (BiB) is a prospective pregnancy and birth cohort study based in Bradford, United Kingdom (UK). The study was established in 2007 to examine how genetic, nutritional, environmental, behavioural and social factors affect health and development during childhood, and subsequently, adult life in a deprived multi-ethnic population. Now that children have reached age 7–11 years, BiB is revisiting study families in the first full follow-up of the cohort [1, 2]. This is exploring how lives have changed for BiB families around the key priority research areas of social and emotional wellbeing, cardiometabolic health, and cognitive and sensorimotor development. Data are collected through multiple approaches, including community visits with families, and data collection with children in schools.

This protocol briefly describes the background of BiB, including existing data, and then the rationale and methods for the new data collection. It provides guidance for other researchers working with deprived and/or multi-ethnic populations to maximise recruitment and retention.

### Born in Bradford – the beginning

BiB was established in Bradford in 2007 [1, 2]. It quickly developed into an applied population health research platform to address the high levels of child ill-health in the city. The city includes some of the most deprived areas in the UK, with high levels of infant mortality, childhood obesity, asthma and disability [3]. The aims were fourfold: 1) to investigate early life determinants of health and ill-health amongst families; 2) to use this evidence to develop, design and evaluate interventions to promote health; 3) to provide a model for integrating research into practice; and 4) to build and strengthen local research capacity in Bradford.

With modest initial funding, BiB had to turn to its local network of midwives, obstetricians, paediatricians and health visitors to recruit families and collect data. This model provided an efficient means of recruitment, and also served to promote ownership of the study amongst local health care providers which, in turn, helped to support our model of translating research into practice. As the children grew up this collaborative approach was extended into schools and local government.

A key decision during the design of the cohort study was to consent parents to linkage of routine health (primary and secondary care) and education records, and to allow the BiB research team to access these records. In collaboration with our local provider of electronic health records across primary care (The Phoenix Partnership, SystmOne) we were able to link 99% of BiB participants to their health record. We also worked with our local authority, Bradford Metropolitan District Council, to link BiB children to their Unique Pupil Identification Number (UPN), allowing us to match 84% with their education record.

### The existing BiB resource

Between 2007 and 2010, the BiB cohort recruited 12,453 women with 13,776 pregnancies at 24–28 weeks gestational age, with 13,858 births (live births and stillbirths). In addition, 3449 of their partners were recruited^a.^^1^ A rich set of data were collected during pregnancy and in the immediate postnatal period [1, 2].

**Fig. 1:**
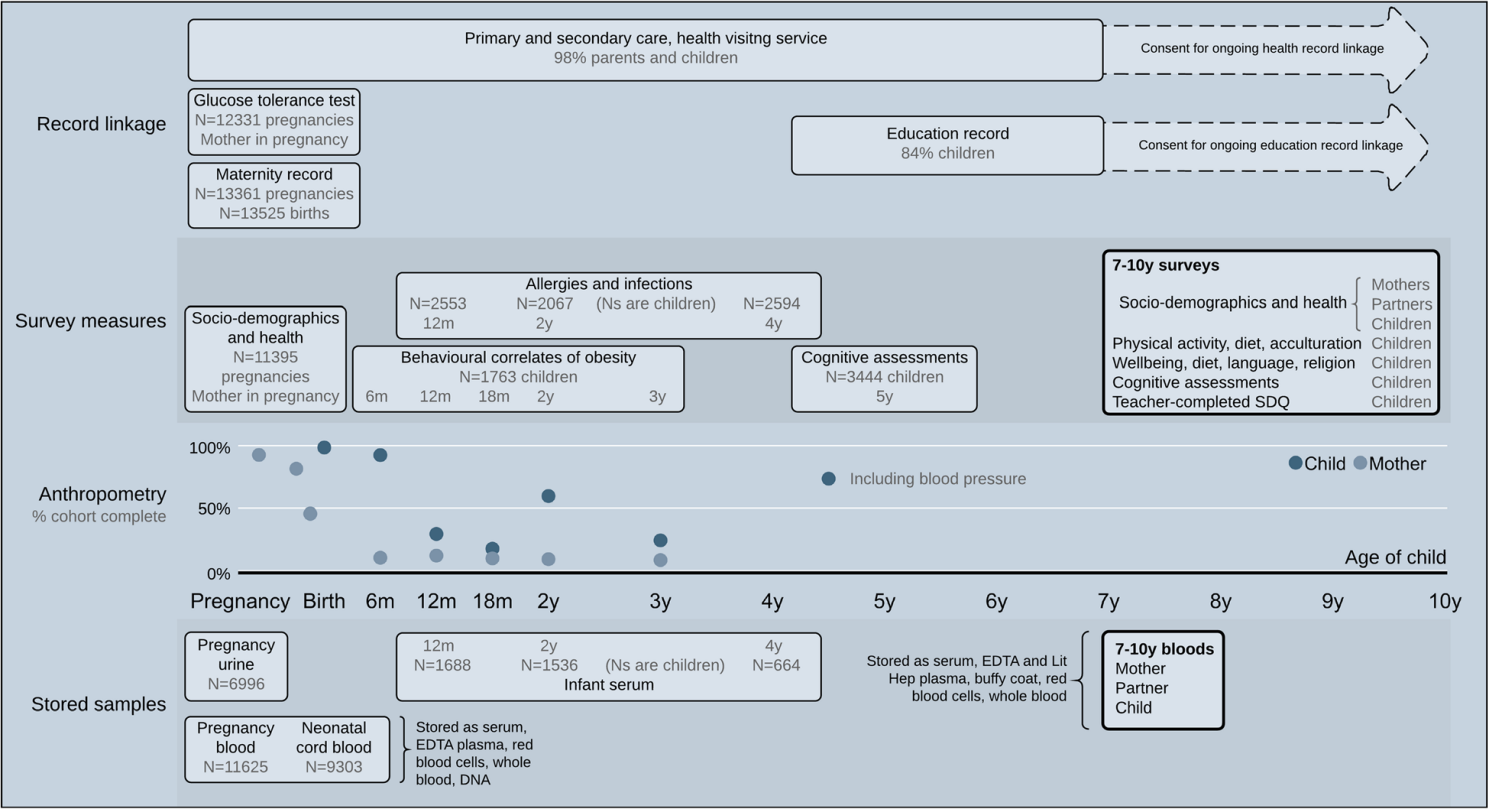
Summary of existing data with the Born in Bradford cohort. Additional File 1 provides full details of all measurements, including assays that have been completed on biological samples to date

Follow-up since then has included growth and blood pressure measures by primary school nurses at age 5–6, [4, 5] whole cohort record-linkage to health and educational data, e.g. [6, 7], detailed bespoke data collection with sub-samples [8–10], and addition of biomarker data using stored biological samples, including genome-wide data, metabolomic data and Deoxyribonucleic acid (DNA) methylation [11, 12]. A summary of data collected can be seen in Fig. 1 and Additional File 1 and full details of recruitment and data collected can be found in the BiB cohort profile [1] and on the BiB website [13].

Analysis of BiB data has provided insights into the factors that affect health and wellbeing in pregnancy and early childhood, including on social and ethnic inequalities in health [14–16], the relationships between maternal gestational adiposity, gestational diabetes mellitus (GDM) and maternal circulating glucose concentrations and fetal growth, birth outcomes, infant and childhood health in South Asian and White European families [4, 5, 17–19], the associations of environmental stressors such as green space and air pollution on health e.g. [20–22], exposures that may influence development of asthma and allergies [23] and the role of genetic variation in human health [12]. BiB data have underpinned more than 100 journal publications; a full list can be found at https://borninbradford.nhs.uk/.

Strengths of the BiB cohort include:

**The diversity of the cohort**. Forty five per cent of families are of Pakistani origin, and half of Pakistani-origin mothers and fathers were born outside the UK [24]. Half of all BiB families are living within the fifth most deprived areas of England and Wales. This provides the opportunity to study the interplay of deprivation, ethnicity, migration and cultural characteristics, and their relationship to child health and wellbeing.

**A life course approach**. The assessment of **multiple-‘omics data** (including genome-wide data, some genomic sequenced data, epigenomic, and metabolomic data) and **lifestyle** in parents and children, with repeat measures of some of these in subgroups and with the current follow-up in the whole cohort supports life course epidemiology. That is, understanding how social, cultural, lifestyle and biological factors interact across the life course and across generations to influence development, health and wellbeing.

**The whole systems, collaborative approach**. BiB involves health and education professionals and a wide range of people in the local community in identifying research priorities and the most efficient and feasible ways of obtaining data to address these. We have integrated our research programme with key organisations within the city including the local authority, clinical commissioning groups, school networks, and voluntary sector organisations. Our goal is to work with these organisations to help set research priorities, influence commissioning of evidence-based services, improve the collection and accuracy of routinely collected data and to raise the bar in the monitoring of standards and the evaluation of interventions to improve health.

**Participant and community engagement**. We have established an active BiB parent governors group, which meets every two months, to help co-produce our research programme. We have a range of activities focused on communicating and disseminating findings to participants and the wider Bradford community, using traditional (birthday cards, newsletters) and social media (Facebook: https://www.facebook.com/BornInBradford, twitter: @bibresearch @bibparents) approaches. We hold regular events to talk with the community about our research, including family science festivals and pop-up events in local shopping centres. We are regularly featured in the press, including a yearly BBC Radio 4 radio programme that features BiB [25].

**The city focus.** The focus on a single city has facilitated engagement with participating families, including traditionally ‘seldom heard’ or ‘under-served’ groups such as families living in more deprived areas. It has also facilitated engagement with key stakeholders and the use of findings in local decision making. BiB has recruited a sample that is largely representative of city’s ethnic and deprivation profile [26].

**Detailed data collection and routine data linkage.** Detailed data collection during pregnancy, including oral glucose tolerance testing which is routinely offered to all pregnant women in Bradford, [18] and repeat ultrasound scans [19], infancy and childhood (the latter supplemented by health and educational record linkage), together with stored biological samples on most participants (Fig. 1), provide a unique opportunity for clinical and public health translational research on maternal and perinatal health and developmental origins of health and wellbeing.

### BiB age 7–11 years follow-up: a resource for future research

#### Multi-method approach

Now that our BiB children are all of primary (elementary) school age, a full follow-up of the children and their families will provide insights into change in their family circumstances, health and wellbeing since birth.

We are employing a multi-method, three-armed approach to collect data from children and parents in community and school settings. This provides an efficient way of collecting data and maintains the close engagement with the whole community that was established at the study’s inception. In our community-based family assessments we conduct face to face visits with consenting parents, either on their own or with their children, in the family home or community venues. We have two independent school-based assessments: one working alongside school nurse teams to collect health-related data with children, and one working with teachers to collect cognitive and wellbeing assessments.

#### Priority research areas

The follow-up data collection focuses on three priority research areas (see below), with differences in ethnicity (including ancestry and migration to the UK) and socioeconomic position being cross-cutting themes. The links between the three focused areas are key to understanding how child and adult health and wellbeing develop.

##### Social and emotional wellbeing

Social and emotional wellbeing is key to the development of healthy behaviours, healthy relationships and educational attainment, prevention of behavioural problems and better mental health [27]. Early work from BiB suggests that consanguineous relationships, mainly seen as a risk factor for congenital anomalies, might simultaneously protect against psychosocial stress [28]. We have also found that Pakistani mothers report feeling more confident about their abilities as a parent and fewer of them adopt a hostile approach to parenting than White British women [29]. Using linked General Practice (GP) data, we have found that Pakistani mothers have a higher prevalence of undiagnosed and untreated depression and anxiety, and that these mothers have children with worse socio-emotional wellbeing at age 4–5 years [6, 14, 30].

The 7–11 years follow-up is supporting in-depth exploration of mechanisms underlying ethnic differences in social gradients in socio-emotional wellbeing, for example by taking into account the process of acculturation and experiences of discrimination (which are likely to differ between parents and children and by the length of time that parents have lived in the UK). This research will provide a strong evidence base for community and family interventions aimed at maximising child and adult well-being, particularly in deprived multi ethnic communities.

##### Growth, adiposity and cardiometabolic health

South Asian adults have a characteristic phenotype of proportionately greater adiposity, increased insulin resistance and higher rates of diabetes and cardiovascular disease compared with white British adults [31–34]. In BiB we have shown that despite being born smaller and lighter, infants of Pakistani-origin mothers have higher total fat mass, which seems in part to be explained by greater circulating fasting and post-load glucose in Pakistani women [17]. Using repeat ultrasound scan measurements we have shown that influences of GDM, fasting and postload glucose emerge prior to the pregnancy stage when gestational diabetes is usually diagnosed [19]. By age 4–5 years, the positive effect of maternal gestational hyperglycaemia seems to have attenuated, though greater maternal early pregnancy adiposity remains associated with greater offspring adiposity at this age [5]. We have also shown that Pakistani women are less likely to experience hypertensive disorders of pregnancy (HDP) compared to white British women, but that HDP is more robustly associated with offspring BP at age 4–5 years in Pakistani compared to white British children [4].

Given the very detailed measurements already available from pregnancy, infancy and early childhood (age 4–5 years), with the follow-up of the whole BiB cohort of children at 7–11 years, we will be able to explore the interplay of socioeconomic, intrauterine and postnatal exposures and molecular mediators on the development of cardio-metabolic traits in South Asian and White British children. We will also be able to explore the relationship of women’s pregnancy experience, and the experience of being parents, on adult cardiometabolic health.

##### Child cognitive and sensorimotor function

Evidence shows that childhood cognitive and visuomotor capabilities are associated with subsequent poorer health and educational outcomes [35–38]. Sensorimotor control is the emergent property that describes the interaction between sensory-perceptual (e.g. visual, haptic) and coordinative (e.g. motor planning) processes required to produce voluntary motor actions [35]. Here we focus on measuring children’s sensorimotor control whilst undertaking three fundamental movement tasks: tracking, aiming and steering. Cognitive abilities may integrate with sensorimotor processes to produce more complex goal directed actions and these abilities refer to higher-level mental processes involved in gaining knowledge, comprehending, remembering, decision-making and problem-solving [36]. We assessed three core components of cognition: Working Memory, Processing Speed and Inhibitory control as others were either likely to be confounded in our multi-ethnic sample (i.e. language) or were unfeasible to measure due to time-constraints (e.g. long-term memory). Evidence shows that abilities in both these domains of function are associated with subsequent poorer health and educational outcomes [37–40]. Children who are socially disadvantaged, including those living in poverty, are at a higher risk of cognitive and sensorimotor deficits [41, 42]. Studies have identified relationships between better social and emotional wellbeing and: manual dexterity among primary school aged children [43] and academic attainment [44] among 4–11 year olds. We have also found that White British children showed higher levels of performance than children from an Arabic education background when moving left-to-right, even on novel tasks, whereas the Arabic education children show the opposite asymmetry [45]. This may relate to exposure to writing and numerical systems that run right-to-left in early childhood before the child enters the UK education system and highlight the role of culture and family on differences in cognitive development.

Measuring child cognition and sensorimotor control at age 7–11 years allows us to relate early genetic, nutritional, environmental, behavioural and social factors to cognitive and sensorimotor capacity in the primary school years. This is essential for identifying those children who are at increased risk and require additional support in early life. As some cognitive and motor capabilities can be improved through specific training [46], this opens up the possibility of interventions that could have a profound impact on physical and mental health in adulthood.

## Aims and objectives

The aims of the follow-up are to investigate the determinants of children’s pre-pubertal health and development, including through understanding parents’ health and wellbeing, and to obtain data on childhood exposures (e.g. maternal, cultural, environmental and economic) that might influence future health. Three key objectives, reflecting the three priority research areas, within which we have identified example research questions are outlined in more detail below. Whilst these objectives are presented separately, in practice they are fully integrated in a programme of work that explores the interrelationships between social and emotional wellbeing, growth, adiposity and cardiometabolic health, and cognitive and sensorimotor development, and seeks to help us understand common mechanisms related to outcomes in these areas.

### Objective 1: to investigate the determinants of child social and emotional wellbeing at age 7–11

#### Example research questions

- How can socioeconomic position be measured in a valid and reliable manner in British South Asian populations?
- Do socioeconomic gradients in childhood and adult wellbeing vary across ethnic, language, generational and migration groups and how might we understand this variation?
- To what extent are good social relationships (e.g. for parents with partners, for children with school friends) protective against adverse effects of low social economic position?
- How do ethnicity and deprivation interact with social exclusion, isolation, loneliness and bullying?
- In the British South Asian population, what roles do social organisation, acculturation, migration, kinship networks and consanguinity play for child wellbeing?
- How are ethnic density and social capital related to child and parental wellbeing and to social and residential mobility? What roles do ethnicity, migration status, consanguineous unions, cultural and kinship ties and socioeconomic position play? What role do acculturation and timing of migration play?

### Objective 2: to identify the determinants of healthy childhood growth, adiposity and cardiometabolic health in children and adults

#### Example research questions

- How do maternal gestational body mass index, GDM and circulating gestational fasting and postload glucose, fasting insulin, and multiple metabolites relate to offspring adiposity, change in adiposity, and cardiometabolic risk factors (blood pressure, glycated haemoglobin, multiple metabolites) in White European and South Asian girls and boys age 7–11 years. How likely is it that any associations represent causal intrauterine effects?
- How do health behaviours, adiposity and cardiometabolic risk factors cluster within children, their parents and families? Does this vary by gender, age, ethnicity, time since migration (for parents), family composition, socioeconomic position or neighbourhood characteristics?
- What are the associations of pregnancy complications with cardiometabolic risk factors in women 7–11 years post-pregnancy? Are these associations independent of socio-economic position and health related behaviours and do they vary by ethnicity?

### Objective 3: to investigate the determinants of child cognitive and sensorimotor development at age 7–11

#### Example research questions

- How do cognitive and sensorimotor processes vary across ethnic and language groups, and as a function of gender and socioeconomic position?
- How are child cognition, behaviour and wellbeing interrelated? Are there differences in cognitive and sensorimotor abilities between children showing socio-emotional and behavioural impairment that further vary by socio-economic strata and between ethnic groups?
- How do early life and parental characteristics and distributions of smoking, alcohol, diet and physical activity influence a child’s cognitive and sensorimotor development?
- How does sensorimotor control change from school entry to age 11 years and how does this map to educational attainment? What factors best predict later cognitive ability and educational attainment?
- How do previous experiences and circumstances affect cognitive and sensorimotor developmental trajectories over the child’s life-course?

## Methods

This section provides a summary of methods employed in each arm of the follow-up. Further information on samples, recruitment and data collection is provided in Additional Files 2-6. Table 1 summarises data that are collected in each of the three arms, with further detail below and in Additional files 2, 3 and 6. All information sheets and consent forms can be accessed via the BiB website: https://borninbradford.nhs.uk/research/documents-data/

**Table 1:** Overview of BiB follow-up data collection activities

	Community-based family assessments ^1^	School-based measurements in BiB children ^1^	School-based whole class cognitive, sensorimotor and wellbeing assessments
Parents			
Parent questionnaire (Time: 40 min. Includes: demographics, home and neighbourhood, socioeconomic circumstances, parent health and health behaviours, physical activity and sedentary behaviour, child health and development, socio-emotional wellbeing, acculturation, diet ^5^, parenting ^5^, child allergies ^5^)	**X**		
Strengths and Difficulties Questionnaire (child behaviour)	**X**		
Height	**X**		
Weight	**X**		
Subscapular and triceps skinfold	**X**		
Waist circumference	**X**		
Bioimpedence ^2^	**X**		
Blood pressure and pulse rate	**X**		
Blood sample (fasting) or buccal swab ^3^	**X**		
Dual x-ray absorptiometry (DEXA) scan ^2^	**X**		
Children			
‘Me and my life’ questionnaire (Time: 15 min. Includes: happiness and health, material wellbeing, family, friends and bullying, school, neighbourhood, demographics, aspirations and acculturation)			**X**
Diet and activity questionnaire (Time: 30 min. Includes: physical activity and sedentary behaviour, determinants of physical activity, diet)	**X**	**X**	
Child-completed computerised cognitive and sensorimotor assessment (Time: 30 min)			**X**
Height	**X**	**X**	
Weight	**X**	**X**	
Subscapular and triceps skinfold	**X**	**X**	
Waist circumference	**X**		
Bioimpedance	**X**	**X**	
Blood pressure and pulse rate	**X**	**X**	
Accelerometry		**X**	
Blood sample (non-fasting) or buccal swab ^3^	**X**	**X**	
Urine sample ^4^	**X**		
DEXA scan ^5^	**X**		
Teacher			
Strengths and Difficulties Questionnaire (child behaviour)			**X**

NOTES^: 1^ Where data items can be collected in either community-based family assessments or school-based measurements we mostly collect data at one time, to provide flexibility and optimise recruitment (some repeat measurements are collected for use in quality control and reliability assessments). ^2^ Excluding women who are pregnant. ^3^ Offered as an option if participant does not want a blood sample to be taken. ^4^ For subsample of children in sub-study on renal function only. ^5^ For subsample only

### A) Community-based family assessments

Community-based family assessments take place at home visits or in community settings (e.g. children’s centres, schools or GP practices). Participants eligible for a DEXA body scan are invited to our clinically equipped “Big BiB bus” mobile clinic. For those we are unable to visit in person or who live outside the Bradford area we are offering telephone/online follow-up. All children and parents who have previously been recruited to BiB are eligible to take part, and adults with parental responsibility for a BiB child who have not previously been recruited are invited to join the study. Each family is sent an invitation letter and three information sheets: ‘Information for families’, ‘Information for children’, and ‘How we use routine information’. Parents provide informed consent for themselves and their child, including consent to future linkage to health and education records. The following data are collected:

**Questionnaires:** Parents complete a self-administered questionnaire on a tablet computer and children complete a short diet and activity questionnaire administered by a BiB Research Assistant. A subsample of parents are asked to complete additional, optional questionnaire ‘modules’ (see Table 1 and Additional File 2).

**Samples and measurements:** We measure height, weight, subscapular and triceps skinfold thickness, waist circumference, bioimpedence, BP and pulse rate, take (non-fasting) blood samples (buccal swabs in those who do not provide a blood sample). We complete DEXA scans and collect urine in subsamples (Table 1 and Additional File 3). Assessments are completed by trained staff following standard operating procedures. We provide the option for participants to receive feedback on some of their results and letters for participants to take to their GP if any results could have clinical implications (see Additional Files 4 and 5 for examples of leaflets).

### B) School-based measurements on BiB children

BiB children who attend a primary school within the Bradford Metropolitan District Council area (141 schools) and are in school years 3–6 (age 7–11 years) are eligible to take part in these assessments. We expect to obtain measurements from at least 90% of BiB children attending Bradford primary schools (total *n* = 11,711), an estimate based on previous work [47]. Information and consent forms for measurements in school are posted to the home address of eligible BiB families and are also provided via school.

For anthropometric (height, weight, skinfold, bioimpedance) and BP measurements, parents are sent an opt-out consent form (consistent with the consent process used for the National Child Measurement Programme, NCMP). For the accelerometry and blood samples, parents provide opt-in consent by completing and signing a consent form for their child which is either returned via a freepost envelope or the child’s school. Prior to each component of the child’s assessment, we ensure that they are comfortable and happy for the measure/sample to be taken. School nurse teams collect height, weight, skinfold thickness (subscapular, triceps), systolic and diastolic BP and bioimpedance, using standard operating procedures. Since 2018, trained BiB Research Assistants work alongside the school nurse team to administer accelerometers and a child-completed diet and activity questionnaire and take a non-fasting blood sample. It is currently too early to predict the response proportion to these measurements.

### C) School-based cognitive and wellbeing assessments in all children

In this arm only, we conduct assessments with the whole class (including children who are in BiB and children who are not part of the study) at the request of teachers in the included schools. Data are collected in three academic school years (3–6, covering ages 7–11 years) in 90 primary schools that have high numbers of BiB participants attending them. Over 24,000 children are eligible to participate, with ~ 9000 of these being BiB children. After schools agree to take part, they provide information sheets and consent forms to parents; parents provide ‘opt-out’ consent.

A team of trained research assistants administer standardised assessments of sensorimotor control [48], working memory, processing speed and inhibition via tablet computers (further information is provided in Additional File 6). Children also complete an online questionnaire (‘me and my life’) which assesses aspects of child wellbeing (see Table 1). Teachers complete an assessment of each child’s behaviour and socio-emotional development using the Strengths and Difficulties Questionnaire (SDQ) [49].

Teachers are provided with a 1 page summary of cognitive assessment results for each child in their class, with a visual summary of results showing the child’s capability relative to other Bradford children in their age group (see Additional File 7).

### Sample size considerations

Our goal is to obtain as high a follow-up rate as possible of our original cohort to maximize the value of our resource. Based on our previous experience we have estimated a 70% response rate, thus we hope to reach at least 9000 of the 13,354 currently active children in the cohort, 8400 mothers, and increase the number of fathers and partners in the cohort to 5000. Based on previous experience we also anticipated that this would not mean these numbers all had complete data. Numbers with blood samples and valid accelerometer data would likely be lower than those with weight and height, for example. Table 2 shows example calculations of minimal effect sizes based on this previous experience. For all calculations we provided minimal effect estimates for a pre-specified sample size in two ethnic groups. For some of these analyses the groups are White British and Pakistani participants (i.e. the two largest most homogenous groups) for others they are White European and South Asian. The rationale for presenting estimates in different subgroups is that previous publications using existing (e.g. baseline) data from BiB have compared associations between either White British and Pakistani (see for e.g. [4, 5, 17]) or White European and South Asian (e.g. [19]) and the objectives demonstrate that future studies are also likely to compare these groups. BiB has been, and will continue to be, used for research questions where the primary aim is not concerned with ethnic differences; statistical efficiency when all ethnic groups are combined in analyses will be greater and the minimal effect sizes possible to estimate smaller than those presented in Table 2. We have used known numbers where data exist (e.g. for birth characteristics) and conservative estimates where data are still being collected. For objective 2 we have determined the minimum effect size detectable with our given / estimated sample sizes to detect, with 85% power, a two-sided *p*-value of ≤0.01 and also of ≤0.0001 because of multiple testing with omics data in this objective. Outcomes for most questions in objective 2 are continuously measured variables and this is reflected in power calculations, which give the minimal difference in outcome means reported in standard deviation (SD) units per SD/category of exposure. For objective 1 most outcomes are binary and for objective 3 a mixture of continuous and binary. There are potentially fewer problems with multiple comparisons in these objectives, given the focus on testing existing theoretical models. For these, minimal effect sizes are presented as relative risks or difference in mean (SD units) per SD/category of exposures for a two-sided p-value of ≤0.05 with 85% power. Table 2 illustrates that we are likely to be able to detect minimal effects of clinical/public health relevance. For detecting differences in effect between two ethnic groups in additive models (i.e. differences in differences in means or in absolute risk for binary outcomes), even restricting these to the smaller groups of White British and Pakistani, we have 80% power at a p-value of 0.05 to detect a minimum effect difference of at least 0.20 SD between groups with continuous outcomes and at least 0.15 difference in absolute risk effects for binary outcomes. For most binary outcomes assessed in multiplicative models we would have 80% power at a *p*-value of 0.05 to detect at least 25% difference in relative risk effects between the two ethnic groups.

**Table 2:** Example power calculations for study objectives

For objective 1: child social and emotional wellbeing at age 7–11					
Group	Exposure	Outcome (binary)	Number^a^	Minimal effect detectable (RR)^c^ *P* = 0.05	
White British	Receipt of means tested benefits	Being bullied (all or some of the time)	3000	1.11	
Pakistani			3500	1.10	
White British	Not managing financially	Child low wellbeing	3000	1.38	
Pakistani			3500	1.36	
For objective 2: healthy childhood growth, adiposity and cardiometabolic health in children and adults					
Group	Exposure	Outcome (continuous)	Number^a^	Minimal effect detectable^b^	
				*P* = 0.01	*P* = 0.0001
White British	Any maternal metabolite from NMR	Offspring tricep skinfolds at age 10	3000	0.06	0.09
Pakistani			3500	0.07	0.10
White European	Gestational diabetes	Offspring systolic blood pressure at age 10	3400	0.11	0.16
South Asian			3850	0.08	0.10
White British	Cord-blood Epigenome wide DNA methylation^d^	Offspring BMI at age 10	400	0.15	0.24
Pakistani			450	0.14	0.23
White European	Hypertensive disorder of pregnancy	Maternal predicted 10-year cardiovascular risk	3400	0.03	0.05
South Asian			3850	0.03	0.06
For objective 3: Determinants of child cognitive and motor development at age 7–11					
Study / group	Exposure	Outcome (binary)	Number^a^	Minimal effect detectable (RR)^c^ P = 0.05	
White British	Not managing financially	Educational problems	3000	1.23	
Pakistani			3500	1.22	
White British	No safe place to play (at baseline)	Motor development delay	3000	1.52	
Pakistani			3500	1.37	
Study / group	Exposure	Outcome (continuous)	Number^a^	Minimal effect detectable^b^	
				P = 0.01	P = 0.0001
White British	Overall Behavioural Difficulties	Motor Skills Test Battery Score	3000	0.10	0.14
Pakistani			3500	0.11	0.16
White British	Specific Difficulties with Externalising Behaviours	Assessment of Sustained Attention (visuo-motor tracking)	3000	0.11	0.15
Pakistani			3500	0.11	0.15
White British	Specific Difficulties with Internalising Behaviours	Assessment of Executive Planning (timed maze tracing)	3000	0.09	0.12
Pakistani			3500	0.17	0.23

a Numbers are all conservative; b minimal difference in outcome (SD units) per 1SD or 1 category of exposure that can be detected with this sample size; c Minimum Risk Ratio detectable for this sample size at *p* = 0.05. Power set to 85% in all of these estimates

### Quality assurance and language

All data collection is administered or undertaken by trained staff using standard operating procedures. Staff collecting data receive training in information governance and safeguarding as well as the assessments/data collection they are responsible for. We have regular training updates and observation of data collection with feedback as appropriate.

During development of materials we conducted interviews and focus groups to ensure that materials are easy to understand and appropriate for our participants. We conducted eight parent focus groups to obtain feedback on the information sheets and to understand parents’ preferences on feedback of research findings. Questionnaires were tested prior to recruitment using think aloud interviews [50]. We interviewed seven parents and over 20 children as they completed draft questionnaires to check understanding of the questions and appropriateness of response options and reworded questions as required.

At baseline, 82% of questionnaires were completed in English, 13% in Urdu, 5% in Mirpuri and 1% in other languages. In the age 7–11 years follow-up we offer questionnaires in English and Urdu and members of the research team speak English, Urdu and Mirpuri. For other languages we work with interpreters to ensure no families are excluded. For the school-based assessments materials are provided in English only as the majority of Bradford primary school children have adequate English skills to understand our study materials. If children have difficulties, research assistants or teachers help with translation and record any assistance that they provide.

### Ethical approval

Ethical approval has been obtained from the National Health Service Health Research Authority Yorkshire and the Humber (Bradford Leeds) Research Ethics Committee for the community-based family assessments and school-based measures (reference: 16/YH/0320) and the school-based cognitive and wellbeing assessments (reference: 16/YH/0062).

## Discussion

The UK has a long history of successful birth/pregnancy cohort studies that have provided a wealth of knowledge in social and health sciences [51]. BiB is one of the latest in this lineage. Its particular importance lies in its composition and setting, covering a multi-ethnic population that has high rates of deprivation. This is the first comprehensive follow up of all families since its inception. It provides the opportunity to contact the children and their parents at a formative stage in their lives – the primary school years - and collect further detailed information about the health, social circumstances and lifestyle characteristics of children living and growing up in Bradford.

BiB is an applied research programme and one of the main goals of the study has been to promote translation of research into practice. Central to achieving this goal has been the success in embedding BiB research assessments into mainstream health (for example, using the NCMP programme as a vehicle for efficiently collecting additional blood pressure assessments) and education practice (for example, incorporating whole school assessments of cognitive and sensorimotor development, and wellbeing). Fostering close links with the schools has provided an accessible and efficient network of recruitment centres, and is proving to be a particularly successful approach to follow-up. As well as promoting collective responsibility for the success of the BiB project, our collaborative approach has also increased receptiveness to the evidence that emerges from BiB.

This inclusive approach is in line with our longer term ambition to establish a ‘city of research’ which unites our multiple cohorts [1, 52] with citizen science, city-wide linked datasets, intervention research and policy engagement. As a result of close integration of our research with organisations within the city, BiB and partners have been able to attract additional investment into the city to implement and evaluate evidence based interventions to improve health outcomes including ‘Better Start Bradford’ [53], a Sport England funded local delivery pilot [54] and the Department for Education Bradford Opportunity Area [55]. This reflects a whole system approach to improving health and well-being and reducing inequalities in Bradford.

We have faced a number of challenges in organizing the follow-up of the whole BiB cohort. A central challenge is the balance of collecting a wide range of relevant data whilst minimizing participant burden and inconvenience. We have responded to pilot feedback by reducing the core questions in our child and parent questionnaires and by providing flexibility in appointment times and settings. To improve efficiency of data collection, analysis and feedback we use an electronic interface and online data entry, but connectivity has been challenging and we have had to use paper collection when online entry has not been possible.

Since its inception we have viewed BiB as a resource for the international research community and detailed data dictionaries and guidance on how to access BiB data are available on our website: https://borninbradford.nhs.uk/research/how-to-access-data/. The follow-up data that we are currently collecting will be merged with existing data and be similarly made available.

Our follow up assessments will enrich our BiB dataset and enable us to contribute important new evidence about the determinants of social and emotional well-being, cognitive development and cardiometabolic health. We plan to apply this knowledge to develop novel interventions to tackle upstream and early life causes of ill-health and inequality. It is one method (of several) that we are using to catalyze our ‘city of research’ concept to promote health and wellbeing by translating evidence into practice and empowering communities, health and public health professionals, community and voluntary groups, educationalists and researchers to work in partnership to discover how best to improve lives for our city inhabitants.

## Additional files

Additional File 1: Summary of existing data by participant group. Table summarising existing data available for the Born in Bradford cohort (PDF 283 kb)

Additional File 2: Overview of questionnaire domains and sources. Three tables summarising questionnaire domains and sources using in the Born in Bradford age 7–11 assessments (PDF 336 kb)

Additional File 3: Procedures for child and adult measurements and samples. Table summarising procedures for child and adult measurements and samples (PDF 193 kb)

Additional File 4: Adult feedback leaflet. Adult Participant feedback leaflet (PDF 1431 kb)

Additional File 5: Child feedback leaflet. Child Participant feedback leaflet (PDF 6015 kb)

Additional File 6: Further information on Primary School Years Cognitive and Sensorimotor assessments. Description of cognitive and sensorimotor assessments (PDF 473 kb)

Additional File 7: Example of Child assessment feedback summary for teachers. Image of child assessment feedback summary for teachers (PDF 192 kb)

## Abbreviations

BiB: Born in Bradford
DEXA: Dual x-ray absorptiometry
DNA: Deoxyribonucleic Acid
GDM: gestational diabetes mellitus
GP: General Practice
HDP: Hypertensive disorders of pregnancy
NCMP: National Child Measurement Programme
SD: Standard deviation
SDQ: Strengths and difficulties questionnaire
UK: United Kingdom
UPN: Unique Pupil Number

## Footnotes

1 Note these numbers differ slightly from our Cohort Profile [1] due to a small number of additional recruitments and further cataloging and ‘cleaning’ of data after publication of that profile
