# Supplementary material for: Growing up in Bradford: protocol for the age 7–11 follow up of the Born in Bradford birth cohort
Source: BMC Public Health. 2019 Jul 12;19:939. doi: 10.1186/s12889-019-7222-2 (PMC6626420; doi:10.1186/s12889-019-7222-2)
Supplement: Supplementary file 1 — Summary of existing data by participant group. Table summarising existing data available for the Born in Bradford cohort (PDF 283 kb) [file 12889_2019_7222_MOESM1_ESM.pdf]

Additional File 1: Summary of existing data by participant group

Table A1.1 Summary of existing data by participant group

	Mother	Child	Father
**Recruited**	12453 mothers (across 13776 pregnancies)	13858 children	3353 fathers
**Pregnancy/birth**			
Socio-demographics and health questionnaire at recruitment	10519 mothers (across 11395 pregnancies)		3287 fathers (across 3387 pregnancies)
Stored biosamples	*Pregnancy bloods:*<br>11625 mothers<br>Stored as serum, EDTA plasma, red blood cells, whole blood.<br><br>*DNA:*<br>10531 mothers (across 11436 pregnancies)<br><br>*Pregnancy urine:*<br>6996 pregnancies	*Cord bloods:*<br>9303 children<br>Stored as serum, EDTA plasma, red blood cells, whole blood<br><br>*DNA:*<br>9158 children<br><br>*Infant serum:*<br>1688 children at 12m<br>1536 children at 24m<br>664 children at 48m	*DNA:*<br>2938 fathers (across 3022 pregnancies)
Blood biomarkers	10685 mothers (across 11625 pregnancies)	7910 children	
Metabolite profiles	*High throughput NMR:*<br>10574 mothers fasted blood samples 24-28 weeks gestation (across 11479 pregnancies)<br><br>*Mass-spectrometry:*<br>3000 mothers fasted blood samples 24-28 weeks gestation, 1000 of which paired with child	*High throughput NMR:*<br>7890 children at birth (cord-blood)<br>1690 children at 12m<br>1536 children at 24m<br><br>*Mass-spectrometry:*<br>1000 children paired with mother	

	**Mother**	**Child**	**Father**
Genome-wide SNPs, sequencing, and DNA methylation	*Genome-wide SNPs:*<br>10531 mothers<br><br>*Exome sequencing:*<br>3005 mothers<br><br>*White blood cell DNA methylation:*<br>1000 mothers on samples taken 24-28 weeks gestation (paired with child)	*Genome-wide SNPs:*<br>9158 children<br><br>*Exome sequencing:*<br>262 children<br><br>*White blood cell DNA methylation:*<br>1000 children taken from cord-blood (paired with mother)	*Genome-wide SNPs:*<br>1283 fathers
**Anthropometry**<br>Counts of participants measured in time periods relative to child age, taken from multiple sources: Primary and secondary care records, health visiting records, National Child Measurement Programme, school nurse visits, and researcher-led clinics.			
Pregnancy booking<br>Pregnancy recruitment<br>Pregnancy 3rd trimester<br>Child birth<br>Child at 1m to 9m<br>Child at 10m to 15m<br>Child at 16m to 21m<br>Child at 22m to 33m<br>Child at 34m to 45m<br>Child at 46m to 69m	11532 mothers<br>10146 mothers<br>5681 mothers<br><br>1282 mothers<br>1495 mothers<br>1217 mothers<br>1136 mothers<br>1049 mothers	 <br><br>13656 children<br>12809 children<br>4074 children<br>2433 children<br>8256 children<br>1049 children<br>10188 children	
**Routine data linkage**			
Glucose tolerance test at pregnancy recruitment	11231 mothers (12331 across pregnancies)		
Maternity data linkage	*Maternity electronic patient record:*<br>13361 pregnancies<br><br>*Abstraction from maternity notes:*<br>10939 pregnancies<br><br>*Ultrasound scans:*<br>13248 pregnancies	*Maternity electronic patient record:*<br>13525 children	
Primary care records	12311 mothers	13776 children	3296 fathers
Secondary care records	12342 mothers	12469 children	

	Mother	Child	Father
Education records		11667 children	
**Child sub-cohort follow-ups**			
Behavioural correlates of obesity survey<br>(6, 12, 18, 24, 36 month follow-ups)		1763 children	
Allergies and infections survey (12, 24, 48 month follow-ups)		2553 children at 12m<br>2067 children at 24m<br>2594 children at 48m	
Cognitive assessments at school entry (4-5 years)		3444 children	
