# Supplementary material for: Growing up in Bradford: protocol for the age 7–11 follow up of the Born in Bradford birth cohort
Source: BMC Public Health. 2019 Jul 12;19:939. doi: 10.1186/s12889-019-7222-2 (PMC6626420; doi:10.1186/s12889-019-7222-2)
Supplement: Supplementary file 2 — Overview of questionnaire domains and sources. Three tables summarising questionnaire domains and sources using in the Born in Bradford age 7–11 assessments (PDF 336 kb) [file 12889_2019_7222_MOESM2_ESM.pdf]

Additional file 2: Overview of questionnaire domains and sources

Table A2.1: Child diet and activity questionnaire domains and sources

Domain	Source	Topics covered
Physical activity and sedentary behaviour	Bespoke	Active travel<br>Sports participation<br>Time outdoors<br>Screen time<br>Activity skills<br>Physical activity enjoyment<br>Knowledge of PA guidelines
	Physical Activity Questionnaire for Older Children (PAQ-C) [1]	Activity in last 7 days
	Parent and Peer Support Questionnaire [2]	Support from friends, parents and teachers to take part in physical activity/sport
Diet	Day in the Life Of… (DILO) questionnaire [3]	Food/drink consumption on previous day
	Bespoke	Normal school lunch<br>Takeaways
Acculturation	Based on Acculturation Rating Scale for Mexican Americans-II for Children and Adolescents (ARSMA) [4] Questions adapted for use with South Asian population.	Clothing<br>Food<br>Holidays celebrated by family/friends

Table A2.2: Parent questionnaire domains and sources

Domain	Source	Topics covered
Demographics	BiB baseline questionnaire	Ethnic background<br>Household composition Pregnancy
Home	Bespoke	Home ownership<br>Number of bedrooms<br>Smartphone access
Neighbourhood	Bespoke	Availability, satisfaction and use of outdoor/green spaces
Socio-economic circumstances	Bespoke. Developed to map to ISCED levels [5]	Highest educational qualification
	National Statistics Socio-economic Classification (NS-SEC) [6]	Socio-economic Classification
	Bespoke	Household income<br>Financial circumstances
Social circumstances	World Values Survey [7]	Trust of people
	Growing Up in Australia survey [8]	Family attachment and affection
	ONS social capital harmonised question set [9]	Voting in elections<br>Belief that can influence decisions affecting local area
Health and health behaviour	Bespoke	Health conditions<br>Medication<br>Smoking<br>E-cigarette use<br>Alcohol consumption (based on chief medical officer guidelines, 2016)
	Millennium Cohort Study [10]	Longstanding illness/disability and impact<br>Drug use
	Personal Health Questionnaire Depression Scale (PHQ-8) [11]	Depression
	Generalized Anxiety Disorder Screener (GAD-7) [12]	Anxiety
	Short form of the General Self-Efficacy Scale (GSE-6) [13]	Self-efficacy
Physical activity and sedentary behaviour	Bespoke	Television viewing<br>Sitting time

	Active Australia Survey [14]	Vigorous, moderate and walking activity time
Child health, development and behaviours	Millennium Cohort Study [10]	Long-term health conditions and impact<br>Diagnoses<br>Wheezing<br>Bed-wetting<br>Hospital attendance
	Parental-Caregiver Perceptions Questionnaire (P-CPQ) in Child Oral Health Quality of Life Questionnaire (COHQOL) [15]	Health of teeth and mouth<br>Impact
	Bespoke	Sleep patterns<br>Puberty
Social and emotional wellbeing	Strengths and Difficulties Questionnaire [16]	Child's socio-emotional and behavioural difficulties
Acculturation	Bespoke questions based on Acculturation Rating Scale for Mexican Americans-II for Children and Adolescents (ARSMA) [4] and acculturation scale [17]	Speaking/watching TV/reading in English/South Asian language<br>Clothing and food<br>Holidays celebrated
Additional question modules for sub-samples of the cohort		
Diet	Children's Dietary Questionnaire [18]	Fruit/vegetable consumption<br>Fat consumption<br>Snack consumption
	Healthy Home Survey [19]	Where meals are eaten
Food insecurity	Short Form of the 18-item U.S. Household Food Security Survey Module [20]	Able to afford food/balanced meals
Physical activity	Parent supporting and controlling practices questionnaire [21]	Family physical activity<br>Influences of parent behaviour Support to do physical activity
	Bespoke	Knowledge of physical activity guidelines
Parenting	Growing Up in Australia [8]	Confidence in parenting<br>Views on child rearing<br>Behaviour to child
Allergies	ISAAC phase III questionnaire 6-7 years [22]	Asthma<br>Allergies<br>Eczema

Table A2.3: Child 'me and my life' questionnaire domains and sources

Domain	Source	Topics covered
Family and home	Bespoke	Satisfaction with home and local parks
	Adapted from Millennium Cohort Study (MCS) Sweep 4 (age 7) [23]	Family relationships
	Bespoke	Languages spoken
Wellbeing and feelings	Child material wellbeing index developed for the Children's Society[24] and socially perceived necessities from the Poverty and Social Exclusion group[25].	Material wellbeing (including access to pocket money, a laptop, holidays, a car, garden)
	Children's Worlds. International survey of child wellbeing. Age 10 questionnaire. [26]	Concern about money
	MCS Sweep 4 (age 7) [23]	Feeling happy, sad, worried
	Bespoke	Feeling unwell, healthy; can work out what to do next when find something hard
Friends, school and activities	Adapted from MCS Sweep 4 (age 7) [23]	Friends, bullying
	MCS Sweep 4 (age 7) [23]	School
	Bespoke	Clubs and activities
Religion and acculturation	Bespoke	Religion
	Based on Acculturation Rating Scale for Mexican Americans-II for Children and Adolescents (ARSMA) [4] Questions adapted for use with South Asian population.	Clothing, Food, Holidays

REFERENCES

1. Kowalski KC, Crocker PRE, Donen RM: **The Physical Activity Questionnaire for Older Children (PAQ-C) and Adolescents (PAQ-A) Manual** In. Edited by College of Kinesiology UoS. Canada; 2004: 1-38.
2. Prochaska JJ, Rodgers MW, Sallis JF: **Association of parent and peer support with adolescent physical activity**. *Research quarterly for exercise and sport* 2002, **73**(2):206-210.
3. Kipping RR, Jago R, Lawlor DA: **Diet outcomes of a pilot school-based randomised controlled obesity prevention study with 9–10year olds in England**. *Preventive medicine* 2010, **51**(1):56-62.

4. Bauman S: **The reliability and validity of the Brief Acculturation Rating Scale for Mexican Americans-II for children and adolescents**. *Hispanic Journal of Behavioral Sciences* 2005, **27**(4):426-441.
5. UNESCO: **International Standard Classification of Education: ISCED 2011.** In. http://www.uis.unesco.org/Education/Documents/isced-2011-en.pdf; 2012.
6. Office for National Statistics. **The National Statistics Socio-economic classification (NS-SEC).** https://www.ons.gov.uk/methodology/classificationsandstandards/otherclassifications/thenationalstatisticssocioeconomicclassificationnssecrebasedonsoc2010 accessed 20/03/2019.
7. World Values Survey. http://www.worldvaluessurvey.org/WVSDocumentationWV6.jsp accessed 20/03/2019
8. Longitudinal Study of Austrialian Children. **Wave 7 Data Dictionary, Growing Up in Australia Wave** [http://www.growingupinaustralia.gov.au/data/datadict/index.html] accessed 20/03/2019
9. Green H, Fletcher L: **Social Capital Harmonised Question Set. A guide to questions for use in the measurement of social capital**. In.: Office for National Statistics, Social and Vital Statistics Division; 2003.
10. Millennium Cohort Study (2006) **Second Survey: CAPI Questionnaire Documentation**. Centre for Longitudinal Studies, University of London. https://cls.ucl.ac.uk/wp-content/uploads/2017/07/mcs3_capi_questionnaire_documentation_final.pdf accessed 20/03/2019
11. Kroenke K, Strine TW, Spitzer RL, Williams JBW, Berry JT, Mokdad AH: **The PHQ-8 as a measure of current depression in the general population**. *Journal of Affective Disorders* 2009, **114**(1–3):163-173.
12. Löwe B, Decker O, Müller S, Brähler E, Schellberg D, Herzog W, Herzberg PY: **Validation and standardization of the Generalized Anxiety Disorder Screener (GAD-7) in the general population**. *Medical Care* 2008, **46**(3):266-274.
13. Romppel M, Herrmann-Lingen C, Wachter R, Edelmann F, Dungen HD, Pieske B, Grande G: **A short form of the General Self-Efficacy Scale (GSE-6): Development, psychometric properties and validity in an intercultural non-clinical sample and a sample of patients at risk for heart failure**. *Psycho-social Medicine* 2013, **10**:doc1.
14. Australian Institute of Health and Welfare (AIHW): **The Active Australia Survey: a guide and manual for implementation, analysis and reporting**. Canberra: AIHW; 2003.
15. Jokovic A, Locker D, Stephens M, Kenny D, Tompson B, Guyatt G: **Measuring Parental Perceptions of Child Oral Health-related Quality of Life**. *Journal of Public Health Dentistry* 2003, **63**(2):67-72.
16. Goodman R: **The extended version of the Strengths and Difficulties Questionnaire as a guide to child psychiatric caseness and consequent burden.** . *Journal of Child Psychology and Psychiatry* 1999, **40**:791-801.
17. Palmer B, Macfarlane G, Afzal C, Esmail A, Silman A, Lunt M: **Acculturation and the prevalence of pain amongst South Asian minority ethnic groups in the UK**. *Rheumatology* 2007, **46**(6):1009-1014.
18. Magarey A, Golley R, Spurrier N, Goodwin E, Ong F: **Reliability and validity of the Children's Dietary Questionnaire; a new tool to measure children's dietary patterns**. *International Journal of Pediatric Obesity* 2009, **4**(4):257-265
19. Bryant MJ, Ward DS, Hales D, Vaughn A, Tabak RG, Stevens J: **Reliability and validity of the Healthy Home Survey: a tool to measure factors within homes hypothesized to relate to overweight in children**. *International Journal of Behavioral Nutrition and Physical Activity* 2008, **5**(1):1
20. Hamilton W, Cook J, Thompson W, Buron L, Frongillo C, Olson C: **Household food security in the United States in 1995: Technical report of the food security measurement project**. In.

Alexandria (VA), US: United States Department of Agriculture, Food and Consumer Service; 1997.
21. Vaughn A, Hales D, Ward DS: **Measuring the physical activity practices used by parents of preschool children**. *Med Sci Sports Exerc* 2013, **45**(12):2369.
22. Ellwood P, Asher M, Beasley R, Clayton T, Stewart A, Committee IS: **The International Study of Asthma and Allergies in Childhood (ISAAC): Phase Three rationale and methods [Research Methods]**. *The International Journal of Tuberculosis and Lung Disease* 2005, **9**(1):10-16.
23. **Child of the New Century. Age 7 Survey. https://cls.ucl.ac.uk/cls-studies/millennium-cohort-study/mcs-age-7-sweep/** accessed 20/03/2019
24. Main G, Pople L: **Missing out: A child centred analysis of material deprivation and subjective well-being**. London; 2011.
25. Main G, Bradshaw J: **Child poverty and social exclusion: Final report of 2012 PSE study**. York; 2014.
26. **Children's Worlds. International survey of children's wellbeing. http://www.isciweb.org/?CategoryID=190** accessed 20/03/2019.
