# Supplementary material for: Growing up in Bradford: protocol for the age 7–11 follow up of the Born in Bradford birth cohort
Source: BMC Public Health. 2019 Jul 12;19:939. doi: 10.1186/s12889-019-7222-2 (PMC6626420; doi:10.1186/s12889-019-7222-2)
Supplement: Supplementary file 3 — Procedures for child and adult measurements and samples. Table summarising procedures for child and adult measurements and samples (PDF 193 kb) [file 12889_2019_7222_MOESM3_ESM.pdf]

Additional file 3: Procedures for child and adult measurements and samples

Table A.3.1: Overview of measurement and sample procedures

Measure	Procedure
Height (parent and child)	Measured by a Research Assistant using a Leicester Height Measure (d=1mm). Measured without shoes, with head placed in the Frankfurt Horizontal Plane.
Weight and bioimpedence (parent and child)	Measured by a Research Assistant using a Seca digital weighing scale or Tanita body composition analyser (d=100g). Measured without shoes and in light clothing.
Triceps and subscapular skinfolds (parent and child)	Measured by a Research Assistant (in community-based family assessments) or the School Nurse team (in school-based measurements) using a Holtain T/W Skinfold Caliper d=0.2mm (or similar). Measured with participant seated. Triceps skinfold measured on left arm and subscapular on left side (left arm removed from clothing for both).
Arm and waist circumference (parent and child)	Measured by a Research Assistant using a Seca 201 tape, d=1mm. Measured with participant standing. Arm circumference measured at mid-point between acromion and alecranon. Waist circumference measured at high point of the iliac crest.
DEXA scan (parent and child)	Lunar i.DEXA total body scan
Blood pressure (BP) and pulse (parent)	Seated and standing systolic and diastolic BP are measured by a Research Assistant using an Omron electronic monitor 705-CPII. Two seated BP and pulse measurements are taken on the left arm with a minimum 2 minute interval between measurements. Two standing BP measurements are then taken.
Blood pressure (child)	In the community-based family assessments: Seating systolic and diastolic BP are measured by a Research Assistant using an Omron electronic monitor 705-CPII. Two seated BP and pulse measurements are taken on the left arm with a minimum 2 minute interval between measurements.<br><br>In the school-based assessments: Seated systolic and diastolic BP are measured on the left arm by the School Nurse team using an Omron HEM-907 electronic monitor. Two seated BP measurements are taken with approximately a 2 minute interval between measurements.

Blood sample (parent and child)	Blood samples are collected by Research Assistants who are trained in phlebotomy and paediatric phlebotomy. Up to 20ml of blood taken (2 x Gel tubes, 2 x EDTA tubes, 1 x whole blood), and an additional tube if a fasting sample is also taken from a parent. Fasting samples are not requested from children.<br><br>Anesthetic spray or cream are offered prior to the blood sample. A maximum of 2 attempts to obtain a blood sample is permitted for each child. Blood samples are transported to the laboratory at Bradford Royal Infirmary for processing within 4 hours of being taken.
Buccal swab (parent and child)	Collected by Research Assistants using Isohelix SK -1S/MS-01 Buccal Swabs. Participants are given the option of providing a buccal swab if they do not wish to give blood samples. Swabs are taken from the inside of the cheek, at least one hour after eating, drinking or cleaning teeth. Buccal swab samples are transported to the lab for freezing at -80C within 24 hours.
Urine sample (child)	Parents are given or sent a sample collection pack and provided various routes to return the sample to suit the parent (prepaid postage, collection via taxi or by researcher, bringing it to an appointment or dropping it off). They are asked to collect the first void in the morning. Urine samples are aliquoted (x3) then stored at -80C.
Activity monitor (child)	ActiGraph GT3X activity monitors are worn on the hip for 24 hours/day over seven consecutive days to measure physical activity and sedentary behaviour. ActiGraphs are fitted to a belt that is fastened securely to the participant on the hip at the level of the anterior superior iliac spine. Research Assistant instructs children on how to place the ActiGraph on themselves, consistently using the same side of the body.

Note: This table provides a summary of procedures. Standard Operating Procedures are available on request.
