# Supplementary material for: Growing up in Bradford: protocol for the age 7–11 follow up of the Born in Bradford birth cohort
Source: BMC Public Health. 2019 Jul 12;19:939. doi: 10.1186/s12889-019-7222-2 (PMC6626420; doi:10.1186/s12889-019-7222-2)
Supplement: Supplementary file 4 — Adult feedback leaflet. Adult Participant feedback leaflet (PDF 1431 kb) [file 12889_2019_7222_MOESM4_ESM.pdf]

Tips for healthy living from BiB research

Get out and enjoy nature

BiB research has shown that being in green spaces (like your local park) can improve your mood and also improve the mood of your children. Try to visit your local park with your children. For maximum health benefits you could do some fun physical activities like walking, scooting or cycling. You will have fun and so will your children!

There are lots of activities happening in your local parks – you can find details of guided walks and visits here: **www.walkingforhealth.org.uk**

Swap some screen time for active games

BiB research has found that most children in Bradford have too much screen time (like watching TV or using a tablet). This can affect their health, learning and wellbeing. It is good idea for you and your children to minimise screen time for entertainment to less than 2 hours a day.

Try playing active games with your children. For alternative activities to screen time for children, visit the Change 4 Life fun generator: **http://media.nhschoices.nhs.uk/change4life/fungenerator/**

More support for healthy living

NHS Livewell (**www.nhs.uk/Livewell**) has lots of information on healthy living.

NHS Change for Life (**www.nhs.uk/change4life**) gives lots of ideas, recipes and games to help you and your family be healthier and happier.

Bradford Beating Diabetes (**www.bradfordbeatingdiabetes.nhs.uk**) is working to reduce Type 2 diabetes in Bradford

Stop smoking support: The NHS has lots of advice online (**www.nhs.uk/livewell/smoking**) or you can contact Bradford Stop Smoking Service on 01274 437700).

If you are worried about your health, make an appointment to see your GP or nurse

If you have any questions you can ask them at the Growing Up Visit or contact the BiB team.

Tel: 01274 383454 Email: borninbradford@bthft.nhs.uk

@BiBResearch Born in Bradford www.borninbradford.nhs.uk

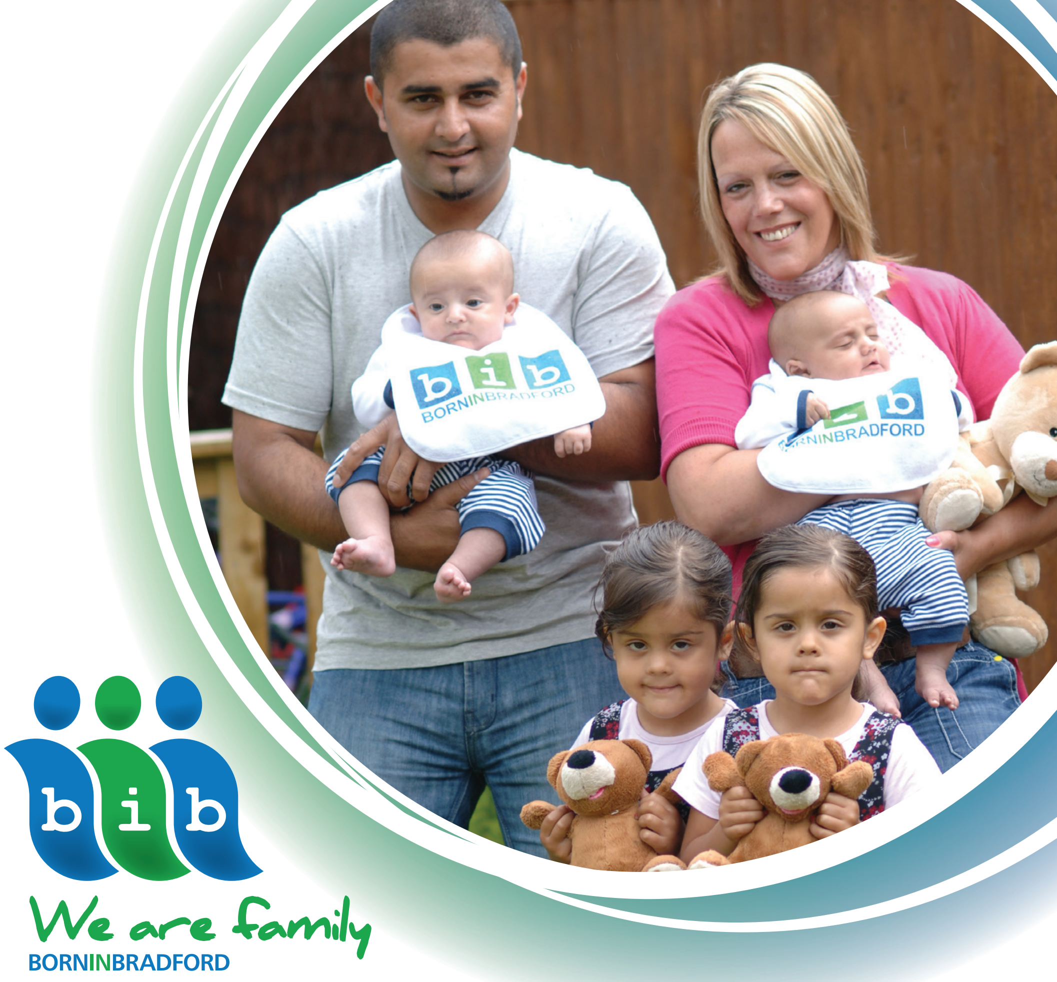

We are family
BORNINBRADFORD

BiB Growing Up Your Results

This leaflet summarises some of the results from your Growing Up visit and explains what they mean. BiB will use these results in our research to help us to understand health and wellbeing. If we find any results that could suggest a health problem, we can give you a letter to take to your GP.

Your BMI

YOUR BMI SCORE

What is BMI?

Body Mass Index (BMI) is a measure that uses your height and weight to work out if your weight is healthy. A BMI above the healthy weight range or too much fat around your waist can increase your risk of serious health problems.

What does your score mean?

BMI	Category	What this means
Lower than 18.5	Underweight	This could be a sign that you're not eating enough or that you may be ill. If you're underweight, your GP can help.
18.5-24.9	Healthy	Keep up the good work.
25-29.9	Overweight	The best way to lose weight is through diet and exercise.
30-39.9	Obese	The best way to lose weight is through diet and exercise and in some cases medication. Contact your GP for advice.

Calculate your BMI

$$\frac{\text{Weight in kilograms}}{\text{Height in metres}^2}$$

Did You Know?

NHS choices says that people from black, Asian and other minority ethnic (BAME) groups have a higher risk of developing some chronic conditions, such as type 2 diabetes.
BAME adults with a: BMI of 23 or more are at increased risk
BMI of 27.5 or more are at high risk

Source: Text adapted from NHS Choices. You can read more about healthy weight at: http://www.nhs.uk/Livewell/loseweight/Pages/BodyMassIndex.aspx

Your Blood Pressure

YOUR BLOOD PRESSURE

What is Blood Pressure?

Blood pressure is a measure of the force that your heart uses to pump blood around your body.

What does your score mean?

High blood pressure is often related to unhealthy lifestyle habits, such as smoking, drinking too much alcohol, being overweight and not exercising enough.

Some medications can cause low blood pressure as a side effect. It can also be caused by a number of underlying conditions and dehydration.

Many different things could have affected your blood pressure today. If you are worried about your blood pressure, you should see your nurse or GP. If your blood pressure result is high, we can give you a letter to take to your GP.

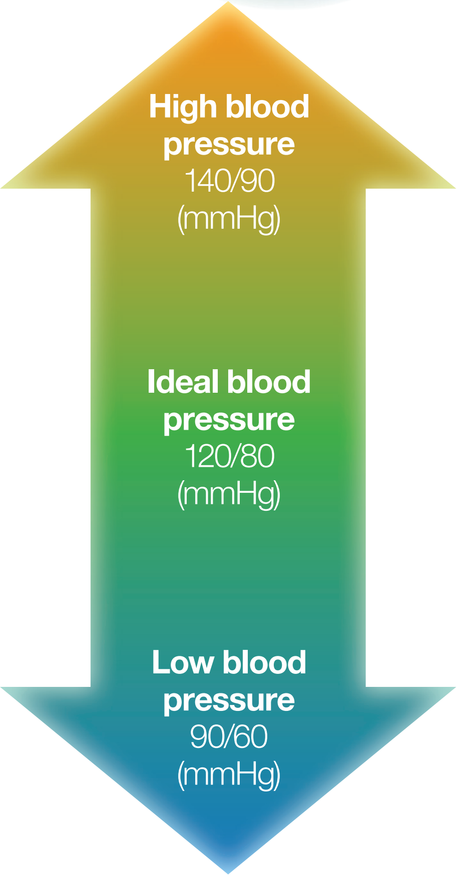

Source: Text adapted from NHS Choices. You can read more about blood pressure at: www.nhs.uk/chq/Pages/what-is-blood-pressure.aspx

Adult feedback leaflet version 1 16.12.16
