# Supplementary material for: Growing up in Bradford: protocol for the age 7–11 follow up of the Born in Bradford birth cohort
Source: BMC Public Health. 2019 Jul 12;19:939. doi: 10.1186/s12889-019-7222-2 (PMC6626420; doi:10.1186/s12889-019-7222-2)
Supplement: Supplementary file 5 — Child feedback leaflet. Child Participant feedback leaflet (PDF 6015 kb) [file 12889_2019_7222_MOESM5_ESM.pdf]

You can find out more about Born in Bradford here:
www.borninbradford.nhs.uk

B	U	W	M	G	H	C	O	L	N	T	A	M	R	S
B	O	G	A	O	L	A	M	H	M	B	H	T	E	Y
A	E	R	A	F	R	W	O	O	L	C	I	T	Y	C
U	B	O	N	C	E	R	B	W	Z	O	G	N	E	G
P	U	W	G	I	F	A	I	Q	D	L	O	P	C	I
L	B	I	I	T	N	O	W	S	C	O	Y	U	O	B
A	P	N	F	Y	A	B	U	S	O	S	E	E	L	N
G	H	G	U	P	W	U	R	M	D	N	S	B	O	T
R	P	U	J	A	L	A	L	A	Y	E	S	C	U	Y
L	R	P	E	R	H	U	B	T	D	M	C	E	R	F
I	K	I	A	K	O	S	C	P	L	F	H	R	I	D
E	H	J	M	K	L	C	Y	M	I	M	O	L	O	C
A	F	A	M	I	L	Y	R	C	C	B	O	R	B	U
C	Y	E	L	B	E	P	O	R	Y	M	L	W	D	O
P	A	C	A	L	H	A	M	B	R	A	D	E	C	L

- City park
- Born in Bradford
- Family
- Bus
- School
- Growing up
- Wool city
- Morrisons
- Alhambra
- Eye colour

@BiBResearch Born in Bradford www.borninbradford.nhs.uk

We are family
BORNINBRADFORD

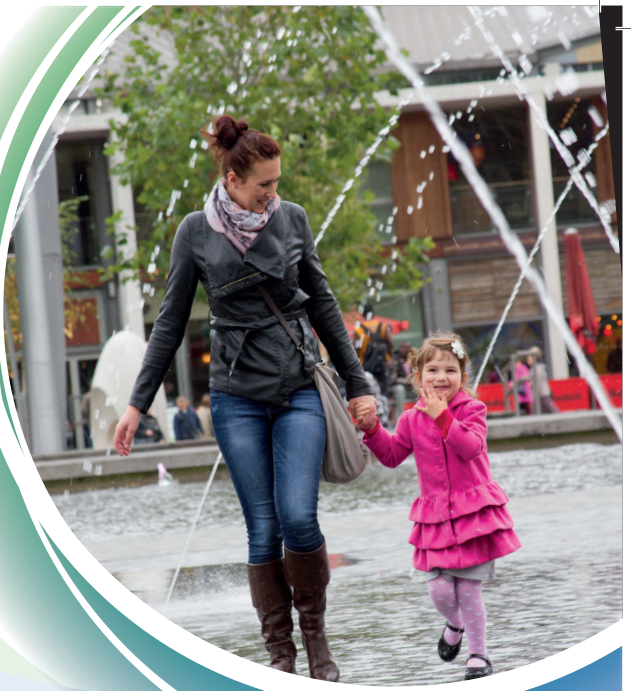

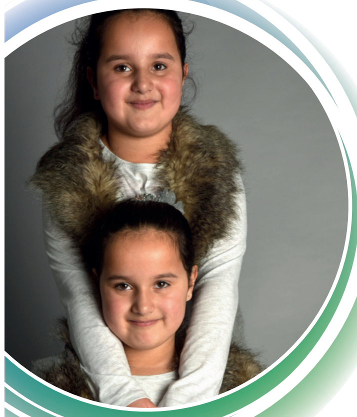

My Born in Bradford Adventure

My Name is

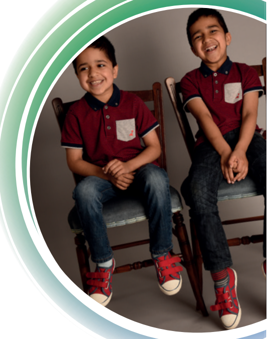

Child feedback leaflet Version 1 16.12.16

BiB_JM_Childfeedback_220117.indd 1-2 06/02/2017 19:21:05

You are part of Born in Bradford

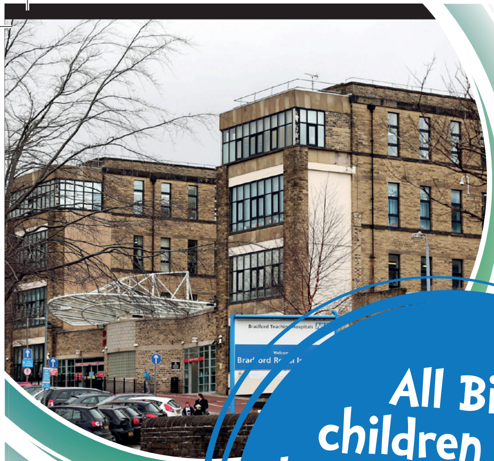

All BiB children were born at Bradford hospital between 2007 and 2011

Now all BiB children are at primary school!

Birth

When you were a baby you helped us to find out that clean air is good for babies'. **Because of this**, now buses in Bradford don't make the air so dirty.

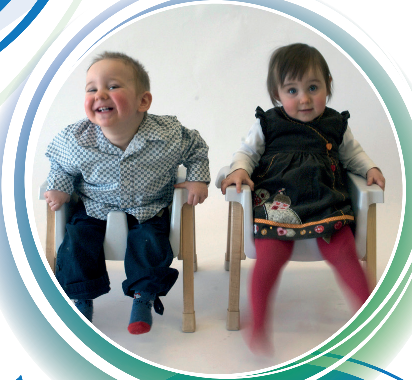

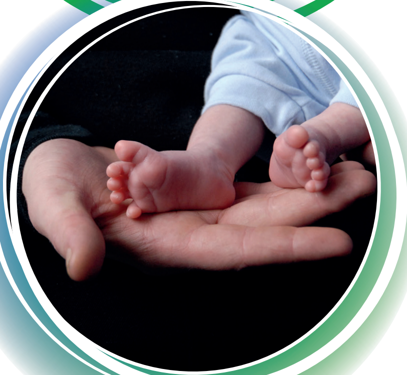

Age 2

When you were a toddler you showed us that doing fun activities with your parents makes you healthy and happy. **Because of this**, now families come and do fun activities in school playgrounds together.

Age 4

When you started school you showed us how to help children do well in class. **Because of this**, now we can help children with their hand-writing which helps their learning!

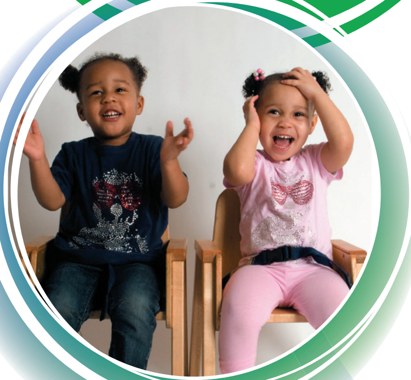

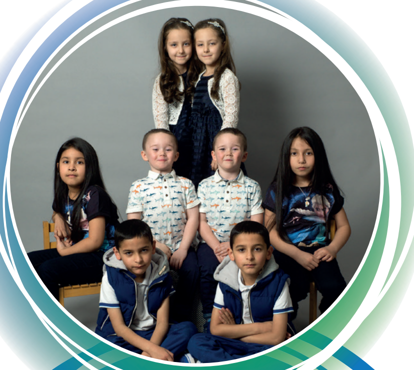

Age 6-8+

Now you are GROWING UP you can tell us all about your life. You are showing us how to help children in Bradford even more!

All About Bradford

Amazing facts about Bradford

The nickname for Bradford is wool city. This is because there were lots of mills making cloth from wool in the 1800s.

Bradford City Football Club were the first League 2 side to reach a major cup final at Wembley in 2013.

William Morrison, who started Morrisons supermarket was from Bradford.

Children in Bradford were the first in England to get free school meals, thanks to Margaret McMillian.

Bradford is the curry capital of Britain and has been for 6 years in a row!

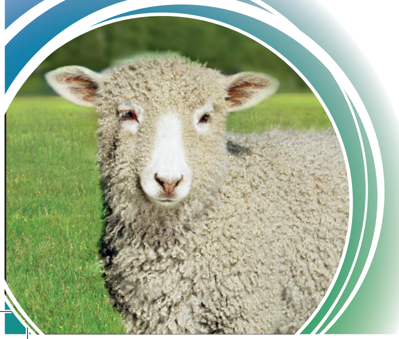

Match up these famous places!

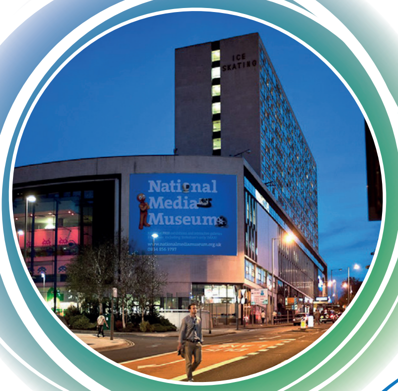

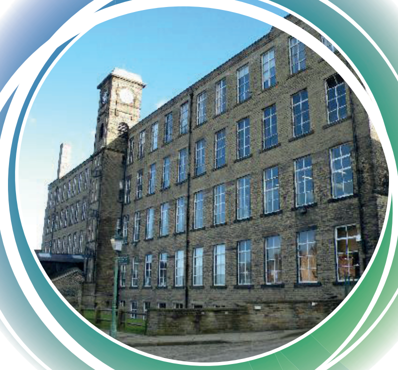

Lister Park

Bradford Industrial Museum

City Park

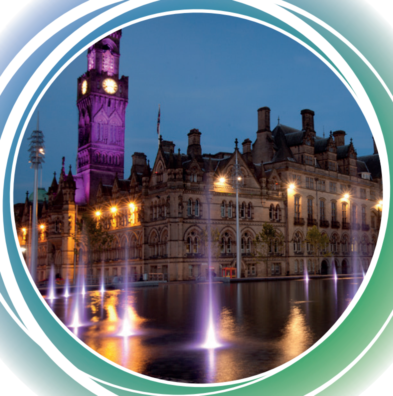

National Media Museum

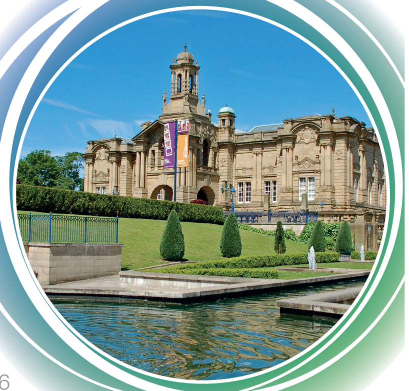

Child feedback leaflet Version 1 16.12.16

BiB_JM_Childfeedback_220117.indd 5-6 06/02/2017 19:21:12

Thank you for taking part in Growing Up! Your answers will be used in our research. Just knowing a little bit about you can help us a lot!

BiB Growing Up

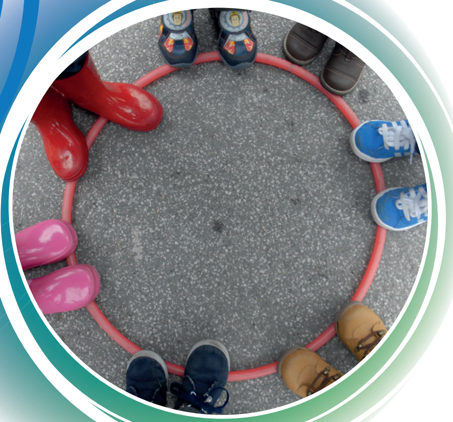

A little bit about me...

I am ______ years old

My eye colour is ______

My height is ______ cm

My hair colour is ______

My weight is ______ kg

Here is a picture of me

Child feedback leaflet Version 1 16.12.16

BiB_JM_Childfeedback_220117.indd 7-8 06/02/2017 19:21:13
