# Supplementary material for: Growing up in Bradford: protocol for the age 7–11 follow up of the Born in Bradford birth cohort
Source: BMC Public Health. 2019 Jul 12;19:939. doi: 10.1186/s12889-019-7222-2 (PMC6626420; doi:10.1186/s12889-019-7222-2)
Supplement: Supplementary file 6 — Further information on Primary School Years Cognitive and Sensorimotor assessments. Description of cognitive and sensorimotor assessments (PDF 473 kb) [file 12889_2019_7222_MOESM6_ESM.pdf]

Additional file 6: Further information on Primary School Years Cognitive and Sensorimotor assessments

The assessments used to measure performance in the following domains were as follows:

Cognition:

Working Memory

Working memory (WM) is a system for temporarily storing information for on-going processing over short periods of time, and is therefore critical for learning. Children identified as having poor working memory abilities have worse educational outcomes across primary and secondary school [1]. Our WM measures covered both simple and complex working memory within the phonological and visuospatial domains. Participants completed three specific computerised tasks (Forward Digit Recall, Backward Digit Recall and Corsi) that make use of widely used methodologies for assessing these aspects of WM performance.

Forward digit recall requires children to listen to sequences of digits presented through headphones, and to remember the digits in the order that they hear them. Participants make responses immediately after each sequence via the tablet screen, by pressing boxes corresponding to the digits they heard, in the order they heard them. The number of digits presented in a sequence increases as the task progresses, with four sequences containing three digits, four sequences containing four digits, four sequences containing five digits, and four sequences containing six digits. No digit is repeated within a sequence. The outcome variable is the proportion correct. **Backward digit recall** has children perform a similar task except they are required to recall the series of digits they hear in reverse order. For backward digit recall, sequence length starts at two digits and increases to five digits (with four sequences at each length). The outcome variable is the proportion correct. In the **Corsi task** children are presented with an array of nine on-screen boxes. A pseudo-random sequence of these boxes then changes colour, one at a time, before returning to its original colour. The participant has to recall the sequence by touching the correct boxes, in the correct order. The number of boxes 'lighting up' in any given sequence increases as the task progresses. Sequence length starts at three and increases to six, with four trials at each length. The outcome variable is the proportion correct.

Inhibitory Control

In order to complete tasks successfully, children need to be able to inhibit irrelevant or distracting information. Inhibitory control is linked to many key developmental outcomes including school engagement and academic success [2]. The **inhibition task** uses a classic flanker design, in which participants are presented with five arrows on the same horizontal line (e.g. <<<<<) and are asked to respond to the direction of the middle arrow (i.e. whether the middle arrow points to the left or to the right). They do so by tapping button icons representing either 'left' or 'right' presented at the bottom of the screen. In this task participants are presented with a mixture of congruent (i.e. the direction of the middle arrow is the same as the flanker arrows: >>>>>), or incongruent trials (i.e. the direction of the middle arrow is the opposite of the flanker arrows: >><>>). Response to incongruent trials is hypothesized to require the participant to exert greater inhibitory control, thus resulting in longer reaction times. The outcome variable is the mean of the reaction times to congruent trials minus the mean reaction times to incongruent trials.

Processing Speed

Processing speed is another core cognitive construct. Improved processing speed enables improved task performance and supports cognitive efficiency [3]. A brief **processing speed task** presents participants with an onscreen array of randomly dispersed triangles and circles. These objects are either red or blue in colour and participants are asked to count the number of red circles they can see within the array and respond with their answer by tapping the appropriate number in a keypad displayed along the bottom of the screen (responses ranged from 1 to 9). Participants complete multiple trials of this task, with their speed of response in milliseconds and number of correct trials recorded as outcome variables.

Sensorimotor Control:

Kinematic analysis of children's sensorimotor control processes are recorded using the Clinical Kinematic Assessment Tool (CKAT) [6,7], which has also been used previously in BiB as part of the Staring Schools study (https://borninbradford.nhs.uk/research/grants/starting-school/). CKAT is a battery of three visuo-manual tests of fundamental sensorimotor control behaviours: tracking, aiming and steering abilities. Detailed descriptions of each task can be found in Flatters et al. [4], with further information on the kinematic variables described in Culmer et al. [5]. All three tasks (see Figure 1) required participants use a handheld 'pen' stylus to interact with 2-D visual

stimuli presented on a tablet computer, with the end point kinematics of the stylus sampled at 120 Hz.

The **Tracking sub-task** assesses participants' ability to continuously track a moving (5mm diameter) target in a sinusoidal figure-8 pattern at three increasing speeds for 3 min, under two conditions: one where a super-imposed guide-path is also present on screen that illustrates the overall movement pattern (reducing predictive demands) and another where it is not (presented first). The ability to produce stable, rhythmic movement patterns is primarily described by Tracking Error (TE): the straight-line distance in millimetres from the centre of a moving target to the tip of the stylus for each sampled time point (i.e. 120 times per second).

The **Aiming sub-task** requires participants to respond as quickly and accurately as possible to seventy-five consecutively displayed 5mm diameter circular on-screen 'targets'. This measures preparatory and online components of participants' responses when making fast, target-directed movements. Once the participant successfully moves to a target then that target disappears and the next target appears on-screen, at the next of one of five onscreen positions (a fixed distance apart) that the sequence cycles through. In other words, every five aiming movements has the participant effectively trace-out the same 'pentagram' path illustrated in figure 1b, before repeating this sequence (i.e. 75 movements equated to repeatedly tracing 15 pentagrams). In the lattermost 25 aiming movements in the 75 move sequence, 6 of these movements involve the target pre-emptively 'jumping' to the next programmed location once the participant moves their stylus within 40 mm of the target, as opposed to waiting until participants arrive in the target area. This un-predictable movement assesses participants' ability to make online corrections to adapt to a shift in target-position during movement. This results in three conditions: Baseline (first 50 aiming movements), Online-Correction (the 6 'jump' trials') and Embedded Baseline (i.e. the remaining standard aiming movements interspersed pseudo-randomly between the 'jump' trials in the final 25 aiming movements of the sequence to make their appearance unpredictable). The movement profile in each aiming movement can be described by the following kinematics: Peak speed (PS) in millimetres per second, Reaction Time (RT), Time to Peak Speed (TPS), overall Movement Time (MT) and Path Length Time (PLT) in seconds, and Normalised Jerk index (NJ). Normalised Jerk Index is a measure of the "smoothness" of an aiming movement, as described in Culmer et al. [5].

The **Steering sub-task** assesses participants' ability to exert precise force control in order to update the trajectory of the stylus, in the course of an on-going multi-component movement in an angular combination of straight-line and curved trajectories. Trials require participant to move their stylus

along a 4mm wide path from a ‘start’ to ‘finish’ zone on the other side of the screen. Two different paths are presented, one per trial, in two consecutive trials. Participants are instructed to try to stay within the path as much as possible and to also to try and stay within a transparent ‘pacing’ box that highlights a portion of the overall path and moves at a fixed speed from the start to finish zone. This box encourages participants to standardise their response speed. Path accuracy (PA) is measured as the mean error in millimetres between stylus position and the centre of the idealised reference path at each sampled point. A pace adjusted Path Accuracy score (PPA) is also calculated, which inflates PA for a trial by the percentage Completion Time (CT) has deviated from the ideal completion time of 36 seconds.

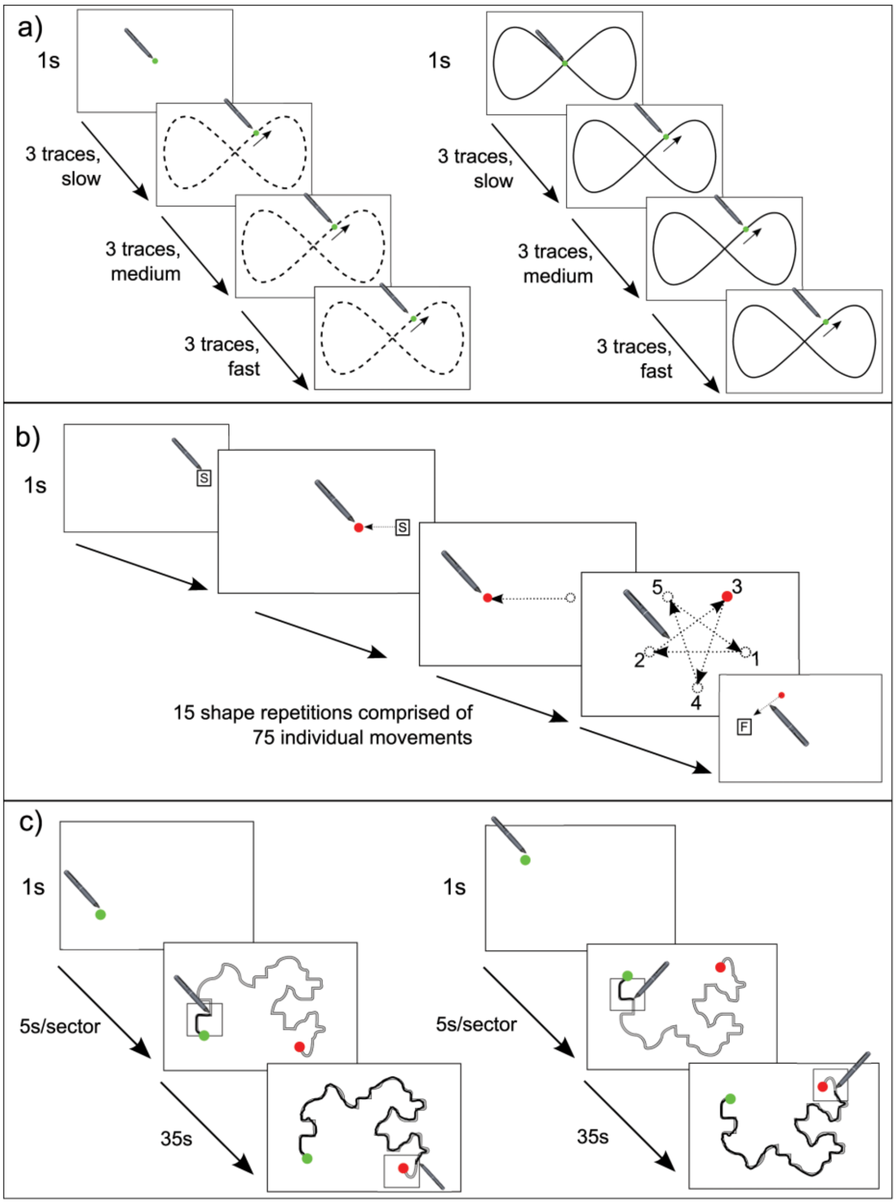

Figure 1. Illustration of the three CKAT sub-tasks, reproduced from Flatters et al. [1]

References:

1. Gathercole, S. E., & Alloway, T. P. (2006). Practitioner Review: Short-term and working memory impairments in neurodevelopmental disorders: diagnosis and remedial support. Journal of Child Psychology and Psychiatry, 47(1), 4–15. http://doi.org/10.1111/j.1469-7610.2005.01446.x
2. Diamond, A. (2013). Executive Functions. Annual Review of Psychology, 64, 135-168. doi: 10.1146/annurev-psych-113011-143750.
3. Kail, R., & Salthouse, T. A. (1994). Processing Speed as a mental capacity. Acta Psychologia, 86, 199-225. doi: 10.1016/0001-6918(94)90003-5.
4. Flatters, I., Hill, L. J. B., Williams, J. H. G., Barber, S. E., & Mon-Williams, M. (2014). Manual Control Age and Sex Differences in 4 to 11 Year Old Children. PLoS ONE, 9(2), e88692. http://doi.org/10.1371/journal.pone.0088692
5. Culmer, P. R., Levesley, M. C., Mon-Williams, M., & Williams, J. H. G. (2009). A new tool for assessing human movement: The Kinematic Assessment Tool. Journal of Neuroscience Methods, 184(1), 184–192. http://doi.org/10.1016/j.jneumeth.2009.07.025
