# Supplementary material for: Growing up in Bradford: protocol for the age 7–11 follow up of the Born in Bradford birth cohort
Source: BMC Public Health. 2019 Jul 12;19:939. doi: 10.1186/s12889-019-7222-2 (PMC6626420; doi:10.1186/s12889-019-7222-2)
Supplement: Supplementary file 7 — Example of Child assessment feedback summary for teachers. Image of child assessment feedback summary for teachers (PDF 192 kb) [file 12889_2019_7222_MOESM7_ESM.pdf]

Additional file 7: Example of Child assessment feedback summary for teachers

Born in Bradford: The Primary School Years

BORNINBRADFORD

[redacted]

Handedness: Right

[redacted]

Date of Test: 24/01/2019

SNAPSHOT

This report should not be used as a screening or predictive tool, rather as a snapshot of the child's development at this point in time.

Room for Improvement → Doing Well

Fine Motor Skills (50 = average, > 50 above average, < 50 below average)

Aiming Score = 97

0 — X — 100

Tracing Score = 100

0 — X — 100

Tracking Score = 96

0 — X — 100

Overall Score = 97

0 — X — 100

Executive Function (50 = average, >50 = above average, <50 below average)

Working memory

FDR score = 94

0 — X — 100

BDR score = 96

0 — X — 100

Corsi score = 96

0 — X — 100

Overall score = 95

0 — X — 100
